# Trimetal-based nanomaterials induced toxicity to plants: Does it differ from the toxicity of mixed and single-element nanoparticles?

**DOI:** 10.1016/j.heliyon.2023.e23178

**Published:** 2023-12-02

**Authors:** Yuchao Song, Mieke van Vlaardingen, Frank Senden, Willie J.G.M. Peijnenburg, Martina G. Vijver

**Affiliations:** a Institute of Environmental Sciences (CML), Leiden University, Einsteinweg 2, 2333, CC, Leiden, the Netherlands; b National Institute of Public Health and the Environment (RIVM), Center for Safety of Substances and Products, Bilthoven, 3720, BA, the Netherlands

**Keywords:** Trimetal-based nanomaterials, Lettuce, Advanced materials, Nanosafety, Metal alloys

## Abstract

Advanced materials comprising multiple metal alloys have made their way into the market. Trimetal-based nanomaterials (TNMs) are an example of advanced materials which have gained significant traction and are now employed in a wide array of products. It is essential to raise the question if the toxicity of advanced nanomaterials like TNMs differs from the joint effects as manifested by exposure to the single component nanoparticles (NPs). To answer this question, a trimetal-based nanomaterial: bismuth cobalt zinc oxide (BiCoZnO) was tested. This TNM had a mass ratio of 90 % ZnO NPs, 7 % Bi_2_O_3_ NPs and 3 % Co_3_O_4_ NPs. Nanoparticle-exposed lettuce seedlings (*Lactuca sativa* L.) showed decreases in relative root elongation (RRE) and biomass production after 21 days of exposure. The 50 % of maximal effective concentration (EC_50_) value of the TNMs for biomass production was 1.2 mg L^−1^ when the exposure period was 240 h. This is of the same magnitude as the EC_50_ values found for ZnO NPs (EC_50_ = 1.5 mg L^−1^) and for the mixture of components NPs (MCNPs) which jointly form the TNMs (EC_50_ = 3.7 mg L^−1^) after 10 d of exposure. The inhibition of plant root elongation by the TNMs was partially (65 %) attributed to the release of Zn ions, with the actual concentration of released Zn ions being lower in TNMs compared to the actual concentration of Zn ions in case of ZnO NPs. It is therefore to be concluded that the concentration of Zn ions cannot be used as a direct measure to compare the toxicity between traditional and advanced Zn-related nanomaterials. The EC_50_ values could be assessed within a factor of two; which is helpful when developing advanced alloy nanomaterials and assessing prospective the effects of trimetal-based nanomaterials.

## 1 Introduction

Over the past few decades, huge amounts of metal-based nanomaterials have been produced and used worldwide [1]. New developments lead to the creation of more complex variations like multiple-metal nanomaterials. Intermetallic nanomaterials (NMs), composed of two or more different metal-based elements arranged in a specific crystal structure [2], have attracted significant attention in catalysis, biomedicine, and energy storage due to their unique properties. These NMs exhibit distinctive attributes, including increased strength, hardness, and improved electronic, optical, catalytic, or magnetic characteristics, multiple-metal based nanomaterials are often preferred over monometallic nanoparticles [3] and have opened up a promising avenue for the development of advanced catalytic materials [4]. The increased efficiency and selectivity of these materials have encouraged further exploration of trimetal-based nanomaterials (TNMs) in catalysis, which are emerging as a new frontier in the field [5]. TNMs offer the potential to further enhancement of the catalytic properties of bimetal-based NMs by introducing a third metal to the system. Although the addition of a third metal can complicate the synthesis and characterization of these NMs, the fundamental principles learned from bimetallic systems can be applied to overcome these challenges. An example of a 10.13039/100012725TNM involves the doping of gold (Au) to FePt NMs supported on a high-surface-area metal oxide [6]. By understanding the effects of electronic and geometric properties on the catalytic performance of TNMs, researchers can design and synthesize novel catalysts with enhanced activity and selectivity for a wide range of chemical reactions. This emerging area of research is expected to have a significant impact on the development of efficient and sustainable catalytic processes for industrial and environmental applications.

The goal of advanced materials development is to meet new needs and overcome existing limitations in various industries. However, it is important to acknowledge that the introduction of new materials also brings about uncertainties with regard to possible impacts on man and the environment [7]. These uncertainties arise due to factors such as limited data availability, complex material properties, potential risks, and unknown long-term effects [8,9]. The advanced nanomaterials can enter terrestrial ecosystems through various pathways, such as disposal of nano catalyst-containing materials, runoff from industrial processes, and accidental spills. Studying their toxicity in terrestrial environments is relevant because it mirrors potential exposure pathways. The bismuth cobalt zinc oxide [BiCoZnO; (Bi_2_O_3_)_0·07_(CoO)_0·03_(ZnO)_0.90_] nanomaterial has been used as an inorganic additive for application in H_2_–O_2_ fuel cells [10] and it has a chemical composition consisting of ZnO NPs, Co_3_O_4_ NPs and Bi_2_O_3_ NPs. It is essential to raise the question if advanced materials like trimetal-based nanomaterials exhibit toxicity different from their main component (ZnO NPs) or to the joint effects as manifested due to exposure to MCNPs (90 % ZnO NPs, 7 % Bi_2_O_3_ NPs and 3 % Co_3_O_4_ NPs). To answer this question, it is crucial to quantify the kinetics of the processes and the reactions that determine the fate of nanomaterials [11]. Dissolution is particularly significant in this regard, as it occurs immediately upon exposure of soluble metal-based nanoparticles to an aqueous environment, resulting in the release of metal ions. The toxicity of metal-based nanoparticles can stem from both the particles and the dissolved ions, or from only one of the two, depending on the kinetics of ion release and the relative toxicity of the ions and particles [12,13]. Particle specific toxicity of Cu NPs has for instance been observed for crustaceans [14], while in the case of ZnO NPs the released ions appear to induce the majority of the toxicity in crustaceans [15]. It has been observed that released Zn ions often have a higher toxicity to plants compared to the particles. This is because the dissolved Zn ions are readily taken up by plant roots and can accumulate in plant tissues, leading to disturbances in cellular processes and essential biochemical pathways [16,17] These examples inform us on the importance of quantifying the ions released from metal-based nanoparticles to understand the toxicokinetics of aquatic suspensions of these materials.

In this study, we quantified the fate and effects of TNMs and the mixture of the three NPs present in this trimetal-based nanomaterial in the same ratio, as well as the fate and effects of the main single component being ZnO NPs following exposure of lettuce (*Lactuca sativa* L.). Being a crucial part of the food chain, the plants are vital in the nutrient cycling and maintaining the function and stability of ecosystems. For this study, lettuce was chosen as one of the model organisms for ecotoxicity testing. The aim of our study is to understand the toxicity of the TNMs, and we therefore investigated: 1) how the crystal structure of the TNMs affects the dissolution of each of the single NPs in ¼ Hoagland solution; 2) the toxicity of suspensions of the TNMs to lettuce and compared the effects induced by the mixture of the component NPs (90 % ZnO NPs, 7 % Bi_2_O_3_ NPs and 3 % Co_3_O_4_ NPs) and the effects induced by the predominant elemental component of the TNM (ZnO NPs); 3) the relative contribution of the Zn ions released from the TNMs to the toxicity of suspensions of TNMs, and to toxicity of the mixture of component NPs (MCNPs) and ZnO NPs at different initial concentrations. In this study we assessed the toxicity of TNMs, MCNPs and ZnO NPs to plant growth by measuring relative root elongation (RRE) and biomass decrease relative to the control every 48 h, for a total exposure time of 480 h. The difference in toxicity between TNMs, MCNPs and ZnO NPs was explained by comparing the Zn^2+^ release as well as by quantifying the toxicity of bismuth (Bi_2_O_3_) and cobalt (Co_3_O_4_) nanoparticles, which are part of the TNMs. Ultimately our study results serve as stepping stones to learn which key principles can be derived from our results and used to make generalized statements about the aspects determining the relative contribution of main component to the toxic impact of advanced materials.

## 2 Materials and methods

### 2.1 Preparation and characterization of NPs suspensions

BiCoZnO NMs (nanopowder, <100 nm), Co_3_O_4_ NPs (nanopowder, <50 nm), Bi_2_O_3_ NPs (nanopowder, 90–210 nm) and Zn(NO_3_) were purchased from the company Sigma-Aldrich (Zwijndrecht, The Netherlands). ZnO NPs (nanopowder, 25 nm, coded NM-110) were obtained from the company PlasmaChem (Berlin, Germany). Suspensions of each nanomaterial were prepared by dispersing materials in 1/4 Hoagland solution (pH 6.0 ± 0.1). The composition of the Hoagland solution is provided in Table S1. Stock suspensions of each material with a concentration of 10 mg L^−1^ were prepared after 30 min of sonication at 60 Hz (USC200T, VWR, Amsterdam, The Netherlands). The size and the morphology of the nanomaterials were characterized by using transmission electron microscopy (TEM, JEOL 1010, JEOL Ltd., Tokyo, Japan). The hydrodynamic size and zeta potential of the tested materials in 1/4 Hoagland solution were determined by means of dynamic light scattering (DLS) on a Zetasizer Nano-ZS instrument (Malvern, Instruments Ltd., Royston, UK). Suspensions of MCNPs were prepared at the same mass ratio of the TNMs (90 % ZnO NPs, 7 % Bi_2_O_3_ NPs and 3 % Co_3_O_4_ NPs) and with the same initial concentrations.

### 2.2 NPs dissolution test

The quantification of the dissolution kinetics of the NPs was conducted in 22 mL glass tubes (15.5 × 150 × 0.8 mm) containing 20 mL of 1/4 Hoagland medium with the initial concentration of the NMs in the suspensions ranging from 0.2 to 20 mg L^−1^. For each of the suspensions of TNMs, Bi_2_O_3_, Co_3_O_4_ and ZnO NPs, 5 mL aliquots were sampled at 6 time points (0.5, 2, 4, 8, 24 and 48 h) in duplicate for the determination of the total concentration of NPs. The remaining suspensions were centrifuged at a speed of 30,392 g for 30 min. After that, 5 mL of the supernatant of the centrifuged samples were sampled and saved for ion concentration determination. All the samples were acidified with a drop of 1 % HNO_3_ and stored in the dark at 4 °C till further analysis. The ion release and total concentrations of the TNMs suspensions and MCNPs suspensions were determined by ICP-MS (PerkinElmer NexION 300D). Given the presence of ligands in the Hoagland medium, it is essential to note that the concentration determined in the test accounts for both free metal ions and those that may form complexes with ligands. To quantify the impact of the mixture of ZnO, Bi_2_O_3_ and Co_3_O_4_ NPs or related ions present in one suspension on the toxicity of the suspension, it is essential to understand the characteristics by which the crystal structure of TNMs impacts the dissolution. To this end, a dissolution test with single element NPs consisting of ZnO, Bi_2_O_3_ or Co_3_O_4_ was performed at the same concentration and equal ratio as present in the TNMs and at the same initial concentration as used in the former tests. The ion release percentage was calculated by means of the following equation:

(1) $$\text{Ion}\;\text{release}\;\text{percentage}=\frac{\text{ICa}}{\text{TCa}}\times 100\%$$

in this equation, IC_a_ is the actual ion concentration determined in the suspensions (mg L^−1^), TA_a_ is the actual total concentration determined in the suspensions (mg L^−1^).

### 2.3 Plants culture and toxicity test design

The toxicity testing of the TNMs was performed in hydroponic cultures. 1/4 Hoagland solution was used as the basic solution for the plants culture. Lettuce (*Lactuca sativa* L.) seeds were purchased from Floveg GmbH (Kall, Germany) and were sterilized by washing the seeds three times with 3 % of H_2_O_2_ in deionized water. The seeds were germinated for four days in a climate room (27 °C, 60 % relative humidity, 16:8 h light/dark cycle). Four seedlings with root lengths between 2 and 3 cm were selected in each Petri dish and exposed to increasing initial concentrations (from 0.2 to 20 mg L^−1^) of TNMs, MCNPs and ZnO NPs (90 % of the nominal concentration of the TNMs) suspensions. The decreases of the relative root elongation (RRE) and biomass production were determined as the toxicological endpoints, which are sensitive to the impacts of external stressors. The exposure medium was refreshed every 48 h and every 48 h the length of the taproot and fresh weight of the seedlings were measured.

The RRE was calculated by means of the following equation:

(2) $$\text{RRE}=\frac{\text{RGs}}{\text{RGc}}\times 100\%$$

in this equation, RG_s_ is the root growth of the plant in the sample solution (cm), RG_c_ is the root growth of the plant in the control solution (cm).

The fresh biomass of seedlings was determined after the seedlings were dried by gentle blotting with a paper tissue. The increase of biomass was calculated according to the following equation:

(3) $$\text{Biomass}\;\text{decrease}=\frac{MG_{c}-{\text{MG}}_{s}}{{\text{MG}}_{c}}\;\times 100\%$$

MG_s_: the fresh biomass of plants in the sample suspension (gram),

MG_c_: the fresh biomass of plants in the control solution (gram).

The toxicity tests were carried out to determine the dose-response curves of the TNMs, MCNPs, and ZnO NPs. In view of the low ion release and low toxicity of Bi_2_O_3_ and Co_3_O_4_ NPs, the actual exposure concentration of Zn ions (ranging from 0.1 to 4 mg L^−1^) was applied as a reference to obtain the dose-response curves of the tested NPs.

### 2.4 Data handling

The actual time-weighted average concentrations (C_TWA_) were used as the metric to describe the concentrations of particles and ions in the exposure medium; this is according to a previous study [18] accounting for the fate dynamics of NPs. The ion concentration was calculated as the time-weighted average concentrations of Zn^2+^ in the TNMs and MCNPs suspensions. The C_TWA_ (Zn ions) of the NPs for each exposure period can be calculated by means of the following equation:

(4) $$C_{\text{TWA}}=\frac{\sum_{n=0}^{N}(\Delta t\;\frac{C_{n-1}+C_{n}}{2})}{\sum_{n=1}^{N}{\Delta t}_{n}}$$

where Δt is the time interval, n is the time interval number, N is the total number of intervals (N = 6), C is the concentration at the end of a specific time interval.

The exposure to particles and dissolved ions induces a decrease in both the root length and biomass of plants and the underlying modes of action of particles and ions are assumed to be independent. The response addition model is therefore used to calculate the relative contributions of each particulate and ionic form to the overall toxicity of the NPs suspensions [19]:

(5) $${{E_{\text{total}}=1-[(1-E}_{\text{ion}})(1-E}_{\text{particle}})]$$

where E_total_ represents either the decrease of RRE or biomass caused by exposure to the suspension of NPs. E_ion_ and E_particle_ are the toxic effects caused by the corresponding ionic and particulate forms (scaled from 0 to 1), respectively. E_total_ and E_ion_ can separately be determined from the experiments and the RRE/biomass decrease caused by the particles (E_particle_) can be calculated by Equation (5). As we based our assessment of overall toxicity on the Zn ion concentration within each NMs suspensions, we have designated the impact of Zn ions as “E_ion_” while considering the collective effects of the remaining components within the TNM or MCNPs suspensions as “E_particle_.” The toxicity evaluation was conducted at the EC_50_ level. Once we established the values for both E_ion_ and E_particle_ in each NMs suspension, we could then proceed to calculate the specific contribution of Zn ions to the overall toxicity.

The value of the EC_50_ for the RRE/biomass decrease was calculated by using the Response-inhibition model in GraphPad Prism 8.0.1 based on the C_TWA_ of Zn ions. E_total_ was quantified by mixture toxicity testing.

### 2.5 Statistics

Statistically significant differences between different exposure concentrations of NPs were analysed by means of one-way ANOVA followed by Turkey's significant difference tests at α < 0.05 using IBM SPSS Statistics 25. Results are expressed as mean ± standard error of 3 replicates.

## 3 Results

### 3.1 Physicochemical characterization of tested NPs

**Fig. 1:**
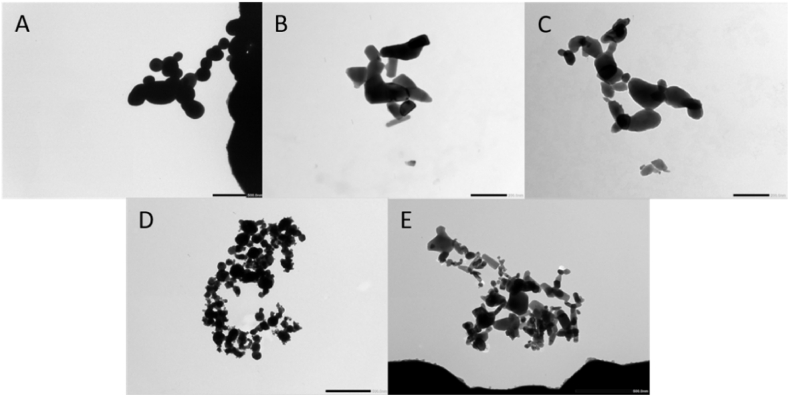
TEM images of Bi_2_O_3_ NPs (A), Co_3_O_4_ NPs (B), ZnO NPs (C), TNMs (D) and MCNPs (E) in the DI water. Scale bars for A, D, and E are 500 nm, while 200 nm for B and C.

The TEM pictures in Fig. 1 showed that the Bi_2_O_3_ NPs (A) were quasi-spherical while the shape of the Co_3_O_4_ NPs (B) and ZnO NPs (C) was platelet. It was observed that the TNMs consisted of particles with irregular shapes (D), which was also observed in the mixture of the three individual metal oxide NPs (E). The DLS results showed that the hydrodynamic sizes of the five NPs in the 1/4 Hoagland medium were significantly greater than their primary particle sizes. This finding indicated that these NPs agglomerated in the test media, and implied that the plant roots were exposed to agglomerates rather than to single particles. The hydrodynamic size of the Bi_2_O_3_ NPs was the highest among all the tested particles at 0 h and it increased relatively fast to a maximum hydrodynamic size of 21,963 nm after 48 h (Table 1). The Zeta-potential of the particles present in the aggregates in the medium was highly negative at different time points and ranged from −16 ± 1.5 to −10.3 ± 0.2 mV.

**Table 1:** Physicochemical characteristics (mean ± standard deviation) of suspensions of the NPs in ¼ Hoagland solution used in this study.

NPs	Size (nm)	Hydrodynamic diameter (nm)^a^		Polydispersity Index (PI)	
		0.5 h	48 h	0.5 h	48 h
BiCoZnO	<100	1889 ± 185	2375 ± 537	1.1 ± 0.04	1.4 ± 0.2
Bi_2_O_3_	90–210	4871 ± 219	21,963 ± 1669	1.9 ± 0.1	0.6 ± 0.02
Co_3_O_4_	<50	835 ± 96	1215 ± 104	0.7 ± 0.1	1.0 ± 0.1
ZnO	25	1630 ± 232	1901 ± 200	0.3 ± 0.1	0.4 ± 0.03
MCNPs	–	2442 ± 617	2560 ± 1260	1.2 ± 0.2	1.2 ± 0.4

### 3.2 Dissolution behaviour of tested NPs in ¼ hoagland

**Fig. 2:**
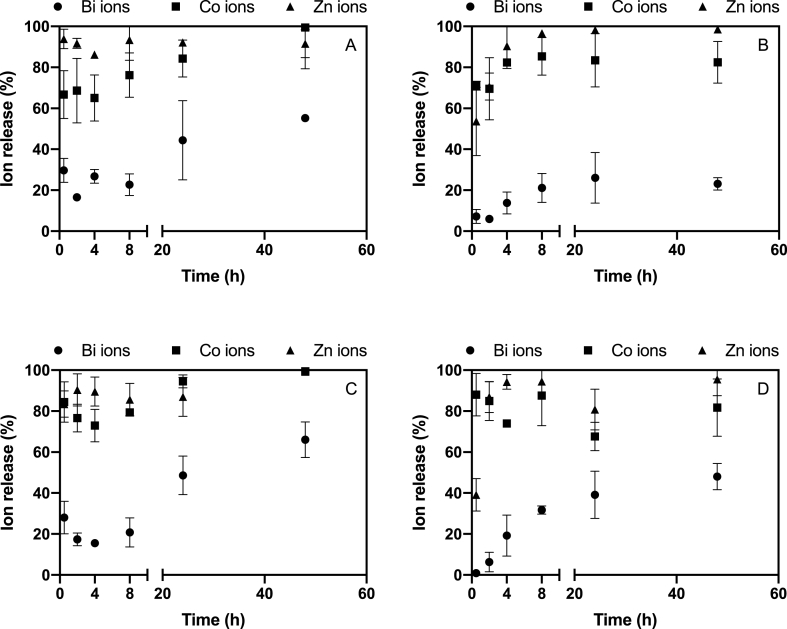
Ion release profiles of the TNMs (A & C) and of MCNPs (B & D) in suspensions at initial concentrations of 2 mg L^−1^ (A & B) and 10 mg L^−1^ (C & D) in the exposure medium over 48 h.

As shown in Fig. 2, the dissolution profile of the TNMs differed from the dissolution profile of MCNPs in the 1/4 Hoagland solution. The release kinetics of ions in both suspensions demonstrated a consistent pattern (Equation (1)), wherein the ion concentration increased over time while accounting for the variability of the initial concentration. The dissolution of both NPs occurred rapidly, requiring approximately 4–20 h to attain a steady state concerning the ion concentrations in the suspensions. The majority of Zn and Co ions were released from both NPs within the first 2–4 h of the experiment. Specifically, the Zn ion percentage reached approximately 90 % during this initial period and remained stable thereafter for two different initial concentrations, except for the suspension with 2 mg L^−1^ of MCNPs (Fig. 2 B). Similarly, Co ions exhibited a similar pattern, with approximately 70 % ion release observed in all NP suspensions. The concentration of Bi released from the TNMs was higher than in the mixture of the individual components jointly composing the TNMs (Fig. 2 A) as approximately 26 % of the Bi present in the TNMs was released over 48 h, whereas 55 % of the Bi_2_O_3_ NPs had dissolved after exposure for 48 h in the mixture of the three NPs (exposure concentration: 2 mg L^−1^). A higher Bi ion percentage (66 %) was found when the initial concentration was 10 mg L^−1^ (Fig. 2C). The percentage of dissolved Co ions in the TNMs suspensions reached 99 %, while it reached 86 % in MCNPs suspensions with the same initial concentration after 48 h of exposure (Fig. 2C & D).

The time-weighted average concentration (Equation (4)) of Zn ions released from the TNMs and ZnO NPs during 48 h were 3.5 mg L^−1^ and 3.1 mg L^−1^, respectively, when the initial concentration was 10 mg L^−1^. These concentrations were higher than the Zn ion concentration present in the mixture of the three individual metal oxide NPs (1.2 mg L^−1^) after the same exposure duration. It was observed that the levels of dissolved Zn ions were comparable between the suspension of TNMs and MCNPs. After 48 h of exposure, approximately 92–99 % of Zn ions were released from the TNMs. Similarly, the ZnO NPs demonstrated a release of 96–99 % of Zn ions after incubation in the presence of the other two NPs in the mixture suspensions at two different initial concentrations. These findings highlight the comparable extent of Zn ion release from both TNMs and MCNPs during the dissolution test. The actual concentrations of Bi and Co ions were found to be lower in MCNPs compared to suspensions of the TNMs at initial concentrations of 2 and 10 mg L^−1^ (Table 2). The Zn ions concentration was 1.2 mg L^−1^ at an initial concentration of MCNPs of 2 mg L^−1^ while it was 3.5 mg L^−1^ in case of TNMs at an initial concentration of 10 mg L^−1^.

**Table 2:** The actual exposure concentrations of released ion and total of TNMs (90 % ZnO NPs, 7 % Bi_2_O_3_ NPs and 3 % Co_3_O_4_ NPs at mass ratio) and MCNPs suspensions in terms of initial concentrations and time-weighted average concentrations.

Nominal Concentration (mg L^−1^)	Initial Measured Concentration (mg L^−1^)																	
	TNMs									MCNPs								
	Bi_(total)_	Bi_(ion)_	Bi_(particle)_	Co_(total)_	Co_(ion)_	Co_(particle)_	Zn_(total)_	Zn_(ion)_	Zn_(particle)_	Bi_(total)_	Bi_(ion)_	Bi_(particle)_	Co_(total)_	Co_(ion)_	Co_(particle)_	Zn_(total)_	Zn_(ion)_	Zn_(particle)_
0.2	0.071	0.019	0.053	0.017	0.013	0.005	0.095	0.089	0.006	0.012	0.001	0.011	0.006	0.004	0.002	0.762	0.659	0.103
2	0.654	0.196	0.458	0.171	0.114	0.058	0.306	0.287	0.019	0.066	0.008	0.058	0.007	0.005	0.002	1.533	1.335	0.198
5	1.386	0.548	0.839	0.300	0.280	0.020	0.711	0.656	0.055	0.006	0.001	0.005	0.023	0.022	0.001	3.710	2.121	1.589
10	2.194	0.959	1.235	0.603	0.457	0.146	1.168	0.972	0.196	0.666	0.007	0.659	0.023	0.022	0.001	7.633	2.933	4.700
20	5.211	2.569	2.642	1.326	0.948	0.378	2.130	1.843	0.287	1.321	0.031	1.290	0.032	0.024	0.008	14.233	3.993	10.240
	Time-weighted average Concentration (mg L^−1^)																	
	TNMs									MCNPs								
0.2	0.021	0.004	0.021	0.016	0.015	0.001	0.097	0.093	0.004	0.004	0.000	0.021	0.005	0.005	0.000	0.727	0.695	0.032
2	0.110	0.040	0.071	0.146	0.119	0.028	0.329	0.312	0.017	0.030	0.003	0.027	0.006	0.005	0.001	1.418	1.367	0.051
5	0.480	0.150	0.330	0.296	0.272	0.025	0.658	0.612	0.045	0.069	0.011	0.058	0.026	0.024	0.002	2.588	2.407	0.181
10	0.881	0.265	0.617	0.587	0.523	0.064	1.247	1.171	0.076	0.143	0.013	0.130	0.031	0.026	0.005	3.877	3.458	0.419
20	1.607	0.523	1.084	1.004	0.853	0.151	2.015	1.911	0.103	0.243	0.036	0.208	0.041	0.027	0.014	4.914	3.680	1.234

### 3.3 Chronic toxicity of the tested NPs to plants

**Fig. 3:**
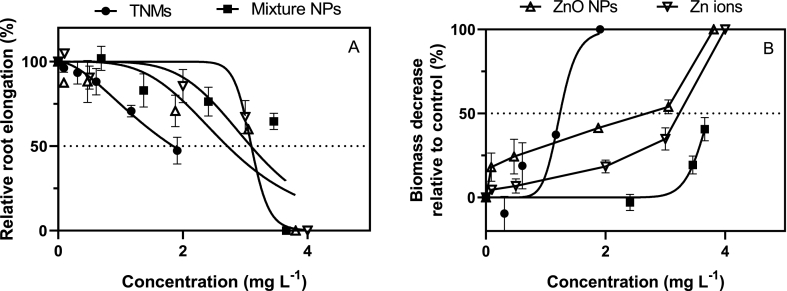
Dose response curves of RRE (A) and biomass decrease (B) induced by TNMs, MCNPs, ZnO NPs and Zn ions suspensions when the exposure time was 240 h. Data are mean ± standard deviation (n = 3).

To determine the toxicity of the TNMs and of MCNPs, the effects on plant growth were determined by measuring the primary root length and fresh biomass every 48 h for a total exposure duration of 21 days. In all treatments, the RRE and fresh biomass of plants decreased as compared to the plants from the control group (Equations (2), (3))), except for the lower concentrations (nominal concentration: 0.2 and 2 mg L^−1^) of the three NPs (Fig. 3 A & B). In the treatment of Bi_2_O_3_ and Co_3_O_4_ NPs (nominal concentration: 0.2–20 mg L^−1^), all the seedling roots length decreased slightly (5.6–19.3 % for Bi_2_O_3_ NPs and −3.0 – 17.5 % for Co_3_O_4_ NPs) while the biomass increased (3.2–43.6 % for Bi_2_O_3_ NPs and 14.3–40.5 % for Co_3_O_4_ NPs) (Table S2) after being exposed for 480 h. Thus, given the low percentage of Bi and Co present in the TNMs, the toxicity of the single metal-based NPs of Bi_2_O_3_ and Co_3_O_4_ can be disregarded in further analyses of their contribution to the toxic effects of the TNMs, except for their interactions within the TNMs.

The values of the C_TWA_ (Zn ions) of the NPs suspensions and the ionic forms causing 50 % inhibition of RRE and fresh biomass (EC_50_) are listed in Table 3. The plants which were exposed to TNMs at concentrations of 10 mg L^−1^ and 20 mg L^−1^ began to die after 240 h. In order to obtain reliable data on the two endpoints under consideration, it is crucial that the maximum exposure duration does not exceed 240 h. The EC_50_ values of TNMs to plant root elongation and biomass production were 1.8 mg L^−1^ and 1.2 mg L^−1^ after 240 h of treatment. ZnO NPs posed similar inhibition of biomass production as the TNMs (EC_50_ = 1.5 mg L^−1^) but induced lower toxicity in case of root growth as the endpoint (EC_50_ = 2.7 mg L^−1^). The TNMs posed higher toxicity with an EC_50_ of 1.2 mg L^−1^ and the EC_50_ of MCNPs was 3.7 mg L^−1^ after 240 h of exposure when the endpoint was biomass decrease. Actually, the EC_50_ of the NPs did not differ significantly when comparing the two endpoints, but there was a difference between the different NPs. The EC_50_ for two assessed endpoints were found to be similar for the ZnO NPs and the MCNPs.

**Table 3:** Comparison of the EC_50_ levels based on C_TWA_ (Zn ions) of suspensions of TNMs, MCNPs, ZnO NPs and Zn ions at the endpoints RRE and biomass decrease (240 h of exposure). EC_50_ values of Bi_2_O_3_ and Co_3_O_4_ NPs are calculated based on nominal concentration. N.D. = not determined.

Types of NPs	Exposure range (mg L^−1^)	EC_50_ (mg L^−1^) (95 % confidence interval)	
		RRE	Biomass decrease
TNMs	0.1–1.9	1.8 (1.6–2.2)	1.2 (1.0–1.4)
MCNPs	0.7–3.7	3.1 (2.5–3.5)	3.7 (3.6–5.2)
ZnO NPs	0.1–3.8	2.7 (2.2–3.3)	1.5 (0.8–2.6)
Zn ions	0.1–4.0	3.1 (2.9–3.4)	3.1 (3.0–3.3)
Bi_2_O_3_ NPs	0.01–1.4	ND	ND
Co_3_O_4_ NPs	0.01–0.6	ND	ND

### 3.4 Relative contribution of Zn ions to the overall toxicity of NPs to plants

**Fig. 4:**
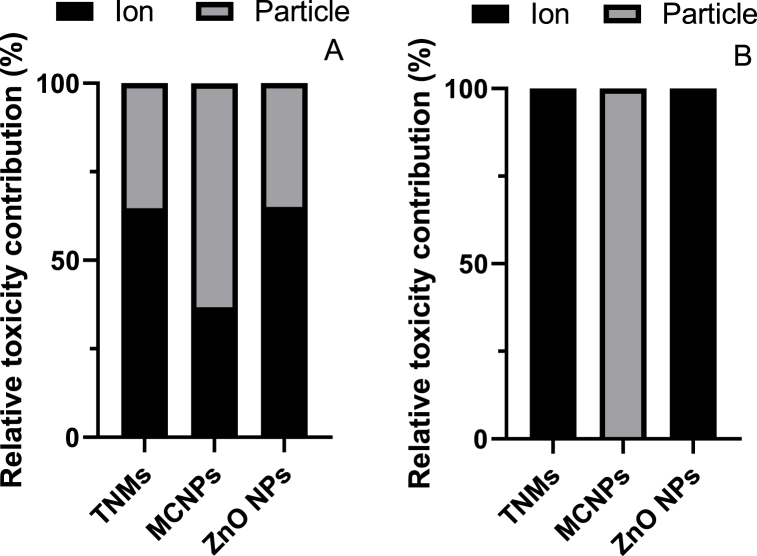
Relative contribution (%) of Zn ions and different particle to toxicity at the EC_50_ level for the endpoints RRE (A) and biomass decrease (B) induced by TNMs, MCNPs, and ZnO NPs when the exposure time was 240 h.

The contribution of Zn ions to the overall toxicity relative to the corresponding particles (Bi and Co NPs) was quantified in all treatments after 240 h (Table 3). The contribution to toxicity for each of the NPs was compared at the level of EC_50_ at two endpoints (Fig. 4 A & B). In the case of RRE as the endpoint of toxicity assessment, Zn ions contributed to 65, 37, and 65 % of the toxicity of the TNMs, the mixture of the component NPs, and the ZnO NPs, respectively. However, when assessing the toxicity in terms of plant biomass production, Zn ions accounted for 100 % of the toxicity in TNMs and ZnO NPs, while no contribution was observed in the toxicity of MCNPs. There is no mechanistic understanding for this calculated result.

## 4 Discussion

Our results illustrated that the TNM released Bi, Co and Zn ions and diminished the growth of the root length and fresh biomass of exposed lettuce plants. The dissolution results showed that the concentrations of released Bi and Co ions from the mixture of the three components forming the TNMs were lower than in case of the TNMs. Bi_2_O_3_ NPs have been reported to be poorly soluble in water [20] and could stay in capillary vessels for a certain period when the vessels were used for medical imaging [21]. This finding is in line with our dissolution results of Bi_2_O_3_ NPs which were less soluble than Co_3_O_4_ and ZnO NPs in MCNPs suspensions. Limited research exists regarding the toxicity of Bi_2_O_3_ NPs to plants. Some studies showed that Bi ions could reduce the root elongation (∼30 % with respect to control) of *Lolium* in 72 h treatment when the plants were exposed to Bi ion concentrations of 242 and 485 mg L^−1^ [22], which are much higher than the concentrations used in this study. On the other hand, Co_3_O_4_ NPs dissolved almost completely (∼99 %) in the TNMs suspensions, while only 85.3–87.6 % of Co ions were released from the MCNPs (Fig. 2).

It is worth noting that the toxic effects of Co_3_O_4_ NPs to plants are reported to vary considerably in the existing literature, depending on the plant species and endpoints used in the test [23]. In the study of Ghodake [24], a negative impact on the root elongation of *Allium cepa* (onion bulbs) was observed in an aquatic medium. When the plants were exposed to Co_3_O_4_ NPs for three days (5–20 mg L^−1^), a massive adsorption of the Co_3_O_4_ NPs on the root surface was observed. Another study showed that nanoparticles (<50 nm) were externally absorbed and did not end up within the plant [25]. This indicates that the toxic impact of nanoparticles might not be due to internal damage, but results from adsorption, more specific blockage of the root tip. In our results we did observe that yellow aggregates in case of exposure to Co_3_O_4_ NPs adhered to the surface of the lettuce roots but the growth of the lettuce seedlings was not influenced by Bi_2_O_3_ nor by Co_3_O_4_ NPs at the exposure concentrations exploited in this study (0.2–20 mg L^−1^) and positive effects of two NPs on the biomass growth were recorded in the treatments.

The impact of bismuth and cobalt ions on the dissolution kinetics of Zn ions in TNMs was found to be minimal (Table 2), thus not affecting the surface properties of the material. This conclusion differs from the findings reported in the a study conducted by Yang [26]. These authors observed an increased dissolution of metals, such as gold, in the presence of bismuth ions. Conversely, Bi_2_O_3_ NPs in MCNPs were found to possess the ability to absorb metal ions due to their high surface area-to-volume ratio. This absorption occurs through ion exchange and the formation of coordination bonds between the metal ions and the Bi_2_O_3_ NPs surface [27]. In our study, the concentration of Zn ions was found to be unaffected by the presence of Bi_2_O_3_ nanoparticles in MCNPs as indicated in Table 2, Table 3 This result is consistent with the ionic Zn concentrations observed in suspensions of ZnO NPs. However, the release of Zn ions was found to be inhibited in the TNMs suspensions, while a higher concentration of Bi ions was also detected. Conversely, the opposite trend was observed in the case of MCNPs. It appeared that the presence of Bi_2_O_3_ NPs in the MCNPs led to the absorption of Zn ions during the dissolution process, resulting in an increased concentration of Zn ions compared to the other two NPs.Although both Bi_2_O_3_ and Co_3_O_4_ NPs did not exhibit negative impacts on plants, the interaction between bismuth NPs and Zn ions in the dissolution process should be taken into consideration.

In general, ZnO NPs are known to be toxic to plants and their toxicity has been attributed to the release of Zn ions from the NPs [28], which can interfere with plant growth and development. The EC_50_ values of ZnO NPs were close to the EC_50_ values of the TNMs at RRE after 240 h in the test, and these EC_50_-values were lower than in case of MCNPs. Our results showed that ZnO NPs nanoparticles induced reduction of root length and biomass of exposed plants. The EC_50_ of ZnO NPs and Zn^2+^ found in this study were much lower compared to EC_50_ values mentioned in other studies [29]. The EC_50_ value (RRE) of ZnO NPs (2.7 mg L^−1^) was also lower than in former studies (5.0 mg L^−1^) of [30]. The possible reason of the lower EC_50_ value of ZnO NPs might be related to the difference in the exposure duration and composition of exposure medium, especially pH and ionic strength are of importance in this respect. Liu [30] determined the RRE after exposing lettuce seedlings for four days, while we employed a longer culture period (10 days) and this difference could account for the difference observed. In this study, we also found that the exposure concentrations of the particulate forms of the tested NPs declined along with time, and time-weighted average exposure concentrations were applied. The TNMs consisted for 90 % out of ZnO NPs and the TNMs as well as the ZnO NPs had similar Zn ion release kinetics and EC_50_ levels. It is noted that the toxicity of ZnO NPs to plants mainly originates from Zn ions [8] and the actual concentration of dissolved Zn^2+^ from the mixture of component NPs and ZnO NPs was higher in comparison to that of the TNMs, which rejected the initial hypothesis that Zn ions are solely responsible for driving the toxicity of TNMs.

Based on our hypothesis, we propose that Zn ions may be the primary factor contributing to the observed toxicity of TNMs, similar to the toxicity associated with exposure to ZnO NPs. Our results have shown that this starting hypothesis should be rejected. The results showed that when the single component NPs were mixed, the Zn ions concentration increased while the toxicity decreased slightly at the same nominal concentration compared to the TNMs (Table 2). Our findings provide further confirmation that Zn ions are indeed toxic to plants. However, it is worth noting that the Zn ions accounted for 65 % of the toxicity observed in plant root elongation, which was higher compared to the toxicity attributed to the mixture of component NPs. The results indicated that the crystal particle structure of TNMs impacts their dissolution and to a little extent also the toxicity. Therefore, we can conclude that the inner structure of the TNMs can influence the characteristics of the nanoparticles as well as the release of ions, resulting in lower Zn ion concentration but higher toxicity to plants. While two endpoints used in the test may respond differently to the NPs, the biomass decrease appeared to be less sensitive compared to RRE. Therefore, when comparing EC_50_ values obtained from different endpoints, caution should be exercised due to inherent variations in sensitivity and response. The dose-response shape differs between the mixtures of the component NPs and the TMNs. It is important to note the disparities in dose-response curves and their slopes, which may arise from the unique structure of TNMs and result in distinct toxicity mechanisms. TNMs receive the necessary attention and consideration separate from other single or mixed element nanomaterials, enabling tailored risk assessment and management approaches [31]. Considering the absence of a unified technological framework for multi-component metal-based NPs, there is a requirement for a categorization and screening method that effectively distinguishes multi-component metal-based NPs from single metal-based NPs. Our results show that the EC_50_ values do not differ largely, thus suggesting that mixture data can be used to predict novel alloy complex toxicity.

Advanced materials have exceptional properties or specific functionalities that differentiate them from the rest of materials. The primary objective of developing advanced materials is to address emerging requirements and overcome existing limitations across diverse industries. However, it is important to acknowledge that the introduction of new materials also brings about uncertainties. To assess the uniqueness and distinct characteristics of advanced materials (TNMs in our case), similarity tools and rankings can be applied. These tools aid in determining if TNMs are significantly different from consisting components NMs and from common materials. Solely assessing the individual elements or mixtures of these component materials gives a magnitude and hence screening opportunities. Even Although there is no significant difference between the EC_50_ of TNMs and ZnO NPs, it cannot be concluded that we do understand the processes on fate and effects induced by TNMs. After all, understanding of the process is not captured which makes it for more depth knowledge inadequate. Therefore we plea that TNMs requires the necessary attention and consideration separate from other single or mixed element nanomaterials, enabling tailored risk assessment and management approaches [31]. By employing tiered approaches, from screening towards in depth knowledge, researchers and regulatory authorities can gain insights into the specific properties and potential risks associated with TNMs. This, in turn, enables targeted risk assessments and the implementation of appropriate management strategies.

## 5 Conclusions

In this nanotoxicology study, different toxicity tests were combined to determine whether there was a difference in toxicity posed between TNMs, the mixture of component of NPs, and ZnO NPs for plants. The TNMs inhibited root growth and biomass production of lettuce and they were more toxic compared to MCNPs at the same mass ratio and exposure dosage after 240 h of exposure. The TNMs had a crystalline structure which reduced the rate of Zn-ion release in comparison to the Zn^2+^ shedding from ZnO NPs. The TNMs did cause higher toxicity to lettuce as compared to the suspension in which MCNPs were used with the same composition and mass ratio of the metal-based NPs. Key principles learned and understood from this study are that ion release from the TNMs is lower compared to the single main component (ZnO) NPs and MCNPs. Furthermore, we learned that the crystalline structure of the TNMs impacts the toxicity, with a suspension of TNMs being more toxic than expected based on the toxicity levels quantified for MCNPs. It is nevertheless also shown that these differences are not large and hence if toxicity data on component of NPs are present, these can be used to predict the alloy complex toxicity within a certainty of a factor of two.

## Data availability statement

Data will be made available on request.

## CRediT authorship contribution statement

**Yuchao Song:** Writing – original draft, Visualization, Investigation, Funding acquisition, Formal analysis, Data curation, Conceptualization. **Mieke van Vlaardingen:** Investigation, Formal analysis, Data curation. **Frank Senden:** Investigation, Formal analysis, Data curation. **Willie J.G.M. Peijnenburg:** Writing – review & editing, Supervision, Resources, Project administration, Funding acquisition, Conceptualization. **Martina G. Vijver:** Writing – review & editing, Supervision, Resources, Project administration, Funding acquisition, Conceptualization.

## Declaration of competing interest

The authors declare that they have no known competing financial interests or personal relationships that could have appeared to influence the work reported in this paper.
