# Supplementary material for: Trimetal-based nanomaterials induced toxicity to plants: Does it differ from the toxicity of mixed and single-element nanoparticles?
Source: Heliyon. 2023 Dec 2;9(12):e23178. doi: 10.1016/j.heliyon.2023.e23178 (PMC10750049; doi:10.1016/j.heliyon.2023.e23178)
Supplement: Multimedia component 1 [file mmc1.docx]

**Supporting Information**

**Trimetal-based nanomaterials induced toxicity to plants: does it differ from the toxicity of mixed and single-element nanoparticles?**

Yuchao Song^1*^, Mieke van Vlaardingen, M^1^, Frank Senden^1^, Willie J.G.M. Peijnenburg^1,2^, Martina G. Vijver^1^

^1^Institute of Environmental Sciences (CML), Leiden University, Einsteinweg 2, 2333 CC Leiden, the Netherlands

^2^National Institute of Public Health and the Environment (RIVM), Center for Safety of Substances and Products, Bilthoven 3720 BA, the Netherlands

***Corresponding author**:

Yuchao Song: [y.song@leidenuniv.nl](mailto:y.song@leidenuniv.nl)

Figures: 1

Tables: 2

Pages: 3

**Table S1.** The chemical composition of Hoagland solution.

| **Chemicals** | **Concentration (mg L^-1^)** |
| --- | --- |
| Ca(NO_3_)_2_·4H_2_O | 945 |
| KNO_3_ | 607 |
| MgSO_4_·7H_2_O | 493 |
| NH_4_H_2_PO_4_ | 115 |
| H_2_BO_3_ | 1.48 |
| Mn(NO_3_)_2_·4H_2_O | 1 |
| Zn(NO_3_)_2_·6H_2_O | 1.19 |
| CuSO_4_·5H_2_O | 0.05 |
| MoNa_2_O_4_.2 H_2_O | 0.02 |
| FeSO_4_.7 H_2_O | 11.1 |

¼ Hoagland solution is prepared by diluting the original Hoagland's solution four times with MilliQ water and pH is measured and adjusted at 6 ± 0.1.

**Table S2.** Effects of Bi_2_O_3_ and Co_3_O_4_ NPs on plants growth during 480 hours of exposure. Data are mean values and standard deviation (SD, n=3).

| Nanoparticles (NPs) | Nominal Concentration  (mg L^-1^) | 48 h | | SD | 240 h | | SD | | 480 h | SD |  |
| --- | --- | --- | --- | --- | --- | --- | --- | --- | --- | --- | --- |
| Relative root elongation (%) | | | | | | | | | | | |
| Bi_2_O_3_ NPs | 0.2 | 101.8 | 4.5 | | | 60.6 | | 1.5 | 81.8 | 8.2 |  |
|  | 2 | 96.8 | 5.1 | | | 69.3 | | 22.1 | 86.3 | 16.8 |  |
|  | 5 | 97.3 | 3.4 | | | 69.0 | | 0.2 | 94.4 | 7.6 |  |
|  | 10 | 96.4 | 6.8 | | | 76.5 | | 0.2 | 96.6 | 15.5 |  |
|  | 20 | 102.3 | 5.2 | | | 85.3 | | 4.9 | 80.7 | 10.7 |  |
| Co_3_O_4_ NPs | 0.2 | 113.3 | 3.1 | | | 86.2 | | 16.4 | 82.5 | 20.1 |  |
|  | 2 | 121.0 | 3.9 | | | 104.1 | | 6.6 | 103.0 | 7.9 |  |
|  | 5 | 108.6 | 16.0 | | | 95.3 | | 9.4 | 101.6 | 0.4 |  |
|  | 10 | 107.5 | 7.2 | | | 89.5 | | 10.4 | 89.3 | 6.2 |  |
|  | 20 | 105.9 | 11.1 | | | 89.4 | | 14.1 | 98.8 | 7.8 |  |
| Biomass decrease (%) | | | | | | | | | | | |
| Bi_2_O_3_ NPs | 0.2 | -31.1 | 2.2 | | | -19.6 | | 18.9 | -43.6 | 5.8 |  |
|  | 2 | -23.7 | 0.9 | | | -30.6 | | 13.9 | -25.3 | 9.0 |  |
|  | 5 | -26.8 | 6.4 | | | 15.2 | | 3.1 | -3.2 | 1.5 |  |
|  | 10 | -51.6 | 13.4 | | | -28.4 | | 3.9 | -21.1 | 3.3 |  |
|  | 20 | -31.1 | 2.2 | | | -42.6 | | 7.0 | -37.6 | 1.5 |  |
| Co_3_O_4_ NPs | 0.2 | -22.1 | 6.6 | | | -22.6 | | 10.1 | -14.3 | 6.6 |  |
|  | 2 | -30.5 | 9.0 | | | -35.1 | | 7.9 | -40.5 | 7.1 |  |
|  | 5 | -16.8 | 6.7 | | | -26.0 | | 9.4 | -21.2 | 10.1 |  |
|  | 10 | -15.3 | 4.7 | | | -5.3 | | 5.8 | -18.9 | 1.2 |  |
|  | 20 | -12.6 | 4.5 | | | -11.4 | | 11.1 | -20.3 | 11.0 |  |


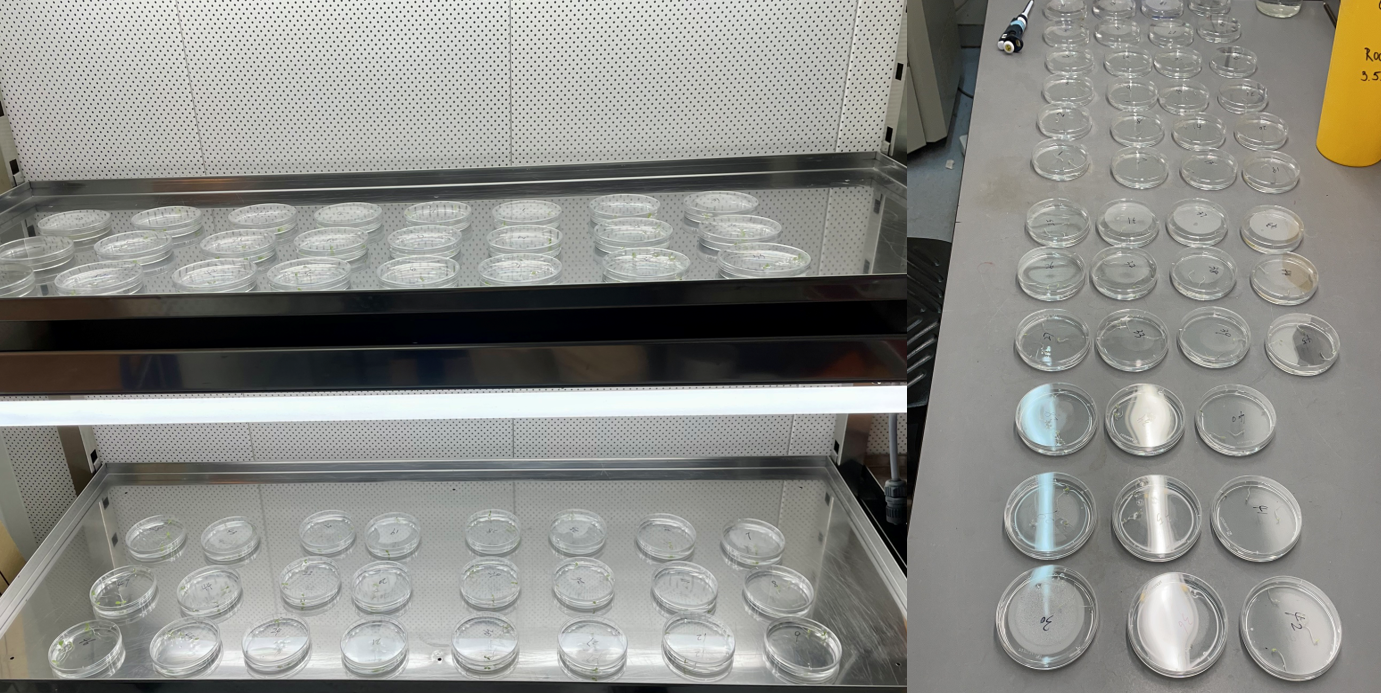


**Figure S1**. Experimental set up of lettuce culture in the climate room.


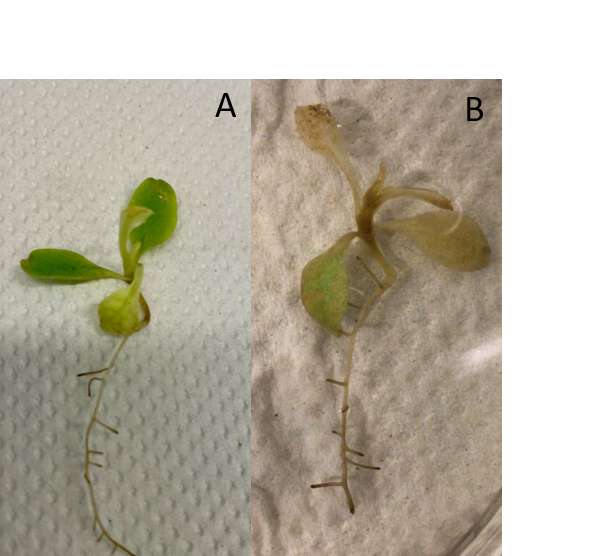


**Figure S2**. Lettuce were exposed to BiCoZnO NMs at 5 mg L^-1^ (A) and 20 mg L^-1^ for 240 hours.
